# Star Trek Offers Insights That Illuminate Actor Engagement in Global Nutrition Governance Comment on "Towards Preventing and Managing Conflict of Interest in Nutrition Policy? An Analysis of Submissions to a Consultation on a Draft WHO Tool"

**DOI:** 10.34172/ijhpm.2020.158

**Published:** 2020-08-24

**Authors:** Vivica I. Kraak

**Affiliations:** Department of Human Nutrition, Foods, and Exercise, College of Agriculture and Life Sciences, Virginia Polytechnic Institute and State University, Blacksburg, VA, USA.

**Keywords:** Conflicts of Interest, Nutrition Governance, Principles, Malnutrition, Healthy Diet, Sustainable Food Systems

## Abstract

This commentary describes insights from Star Trek’s fictional television series to understand how state and non-state actors address conflicts of interest (COIs) through global nutrition governance. I examine the findings of Ralston and colleagues for 44 state and non-state actors who responded to the World Health Organization’s (WHO’s) consultation for a COI risk-assessment tool, developed for member states to engage effectively with non-state actors to address malnutrition in all forms. Star Trek reveals that actor engagement is inevitable in a shared universe. The Prime Directive is a non-interference principle reflecting a moral commitment to reduce harm, respect autonomy and protect rights. Engagement principles are relevant to all actors who influence nutrition policies and programs, and must be held accountable when their actions undermine healthy and sustainable food systems. Certain actors use COI to justify non-engagement with commercial actors yet competing interests, biases, corruption and regulatory capture are distinct challenges to manage. Finally, Star Trek’s characters serve as allegories to understand actors’ motives and actions to promote healthy and sustainable food systems. Unlike non-state actors, states are legally required to achieve their commitments and targets in the United Nations’ (UN) Decade of Action on Nutrition (2016-2025) and Sustainable Development Goals (SDGs) 2030 Agenda.

In 2012, the World Health Organization (WHO) was directed by the World Health Assembly to develop a risk-assessment tool to enable member states to safeguard nutrition policies and programs from conflicts of interest (COIs),^1^ and achieve the WHO’s goal to ensure effective nutrition actions to end malnutrition in all forms by 2030.^2^ Malnutrition is caused by “eating too little food, too much food, the wrong combination of foods, foods with no or little nutritional value, and foods contaminated with disease-causing microbes.”^2^ After an extensive review, the WHO released the COI risk-assessment tool (Table 1)^1^ to guide state’s decisions to engage effectively with non-state actors and promote healthy and sustainable food systems.

**Table 1:** WHO’s Risk-Assessment Tool to Enable Member States to Effectively Engage With Non-state Actors

**Step**	**Objective**
1. Determine the rationale for engagement	1. Clarify the public health nutrition goal
2. Profile and perform due diligence and risk assessment	2. Understand clearly the risk profile of the external actor and the engagement
3. Balance the risks and benefits	3. Analyze the risks and benefits of the proposed engagement based on impacts
4. Risk management	4. Manage the risks based on mitigation measures and develop a formal engagement agreement
5. Monitoring, evaluation and accountability	5. Ensure that the engagement has achieved the public health nutrition goals and decide to continue or disengage
6. Transparency and communication	6. Communicate the engagement activities and outcomes to relevant audiences

Abbreviation: WHO, World Health Organization. Source: WHO.^1^

In 2017, the WHO held an online consultation about the tool developed for states to protect national nutrition policies and programs, and minimize the influence of actors who prioritize commercial interests over human rights and public interests to address malnutrition.^1,3^ Ralston et al^3^ describe an insightful policy analysis of 44 submissions from five distinct groups of actors about the COI risk-assessment tool that revealed how actors understand COI in global nutrition governance. Respondents included member states (n = 6); civil society or public-interest organizations (n = 12); academic researchers (n = 7); commercial-sector entities (n = 14); and United Nations (UN) and other inter-governance bodies (n = 5). Table 2 defines COI and other terms used in this commentary.

**Table 2:** Key Terms Defined

**Term**	**Definition**
Bias	A tendency to support a belief, evidence or favor a position that is consistent with how one thinks or that aligns with one’s values. The WHO background documents defined it as: an influence that impedes the impartial consideration of a question or issue that results in an outcome that favors a particular view or interpretation for decision-making. Intellectual bias occurs when there is the potential to support a specific point of view that could unduly affect an individual’s judgment about a decision.
Competing interest	Divergent interests or different ideological positions on an issue addressed in the policy-making process that may not necessarily imply inappropriate conduct.
Conflict of interest	A situation where there is potential for a secondary (vested interest in the outcome of the government’s work in the area of nutrition) to unduly influence … either the independence or objectivity of professional judgement or actions regarding a primary interest (related to the government’s work). An *individual* COI is a private interest such as financial, personal, or other non-governmental interest or commitment that might interfere with the government’s public health nutrition goals. An institutional COI is a situation where the interests of non-state institutions…economic, commercial or financial (that) are not aligned with the government’s public health policies.
Conflicting interests	A conflict “between” two or more actors who have different opinions or positions on an issue and are opposed to each other. This concept is inherently different from COI that represents a conflict “within” a person or institution between the primary interest and secondary interest of that institution or individual.
Corruption	The misuse or abuse of authority by public officials.
Global nutrition governance	The process by which impact on nutrition by non-nutrition policies (ie, education, employment, health, environment and trade) is leveraged or mitigated by a network of actors. Actors may influence events in global nutrition and work to improve nutrition outcomes through convening, agenda setting, decision-making, implementation and accountability. Actors may be organizations or consortia that form a platform to influence or coordinate actions.
Regulatory capture	A systematic bias in decision-making whereby a government regulatory agency’s primary obligation (regulation to protect and promote public health) is subordinated by a secondary interest (preferences of the regulated party), resulting in decisions that are directed away from the public interest and toward the interests of the regulated industry, by the intent and action of the industry.

Abbreviations: WHO, World Health Organization; COI, conflict of interest. Sources: WHO,^1^ Ralston et al,^3^ Friel et al,^5^ and Busse et al.^18^

Diverse actors influence nutrition policies and programs, food systems, and population health.^4-6^ Friel et al^5^ define *global nutrition governance* as “the process by which impact on nutrition by non-nutrition policies (ie, education, employment, health, environment and trade) is leveraged or mitigated by a network of actors.” They identified three prevailing global nutrition governance challenges: achieving policy coherence across many government sectors that affect nutrition; clarifying actor responsibility for nutrition; and developing effective mechanisms to hold all actors accountable for actions that impact nutrition.^5^ This commentary explores insights from Star Trek’s fictional television series to understand the motives and actions of state and non-state actors who responded to the WHO consultation. I examine Star Trek’s governance and characters to discuss nutrition-related COI and other governance challenges that may hinder the UN and WHO goals to address malnutrition by promoting healthy and sustainable food systems.

## Star Trek: The Federation, Prime Directive, and Engagement

Millions of people worldwide grew up watching Gene Roddenberry’s fictional Star Trek that spanned seven television series (ie, The Original Series, Next Generation, Deep Space Nine, Voyager, Enterprise, and Picard) and 12 feature movies from 1966 to 2020.^7^ Star Trek’s episodes presented ethical dilemmas as futuristic situations for viewers to examine contemporary political, economic and social issues to show a more optimistic future in a post-capitalist society.^7-9^ Star Trek’s philosophy of Manichaeism (principles and actions deemed good or evil) have been used to analyze issues including human rights violations; and political, legal, medical and scientific challenges faced by people living on the planet Earth during the 21st century.^9-11^

Star Trek: The Original Series (1966-1969) portrayed crew members on the USS Enterprise starship in the 23rd century with the mission to seek out new life and civilizations throughout the galaxy, following a recovery from a third world war on Earth and technological developments that enabled space exploration.^7,9^ The lead characters reflected Plato’s moral dimensions of the human soul: Captain James Kirk (*spirit*), Mr. Spock, a half-human, half-Vulcan (*reason*), and Dr. McCoy (*emotion*) who advanced social justice.^7,8^ Star Trek: The Next Generation Series (1987-1994) through Star Trek: Picard (2020) portrayed different captains, crews and starships set in the 24th century.^7,9^ Captain Jean-Luc Picard has been described as “humanity’s conscience and an exemplar of moral autonomy.”^12^

The United Federation of Planets (Federation), of which humans on Earth were a founding member, is Star Trek’s interstellar governing body led by Starfleet Command who follow a governing Constitution.^7,9,12^ The Prime Directive was a moral philosophy and Federation principle that required Starfleet Officers to not interfere with the internal and natural development of other civilizations encountered during their galaxy travels.^12^ The Prime Directive reflected “a consequentialist commitment to reduce harm and a Kantian commitment to respect autonomy, free will and protect citizens’ rights.”^8,12^

The Federation’s governance structure included a Council, President and Supreme Court that parallel the UN System created and reformed during the 20th and 21st centuries to maintain peace and security, promote international cooperation, and harmonize the actions of nation states worldwide. In this commentary, the UN System agencies and other inter-governance bodies represent the Federation and Starfleet Command. The UN System has more than 30 organizations, including the WHO and Food and Agricultural Organization (FAO), which provide guidance to improve nutrition security, food systems and health.The Next Generation’s Captain Picard often said “engage” to initiate new missions^7,12^ because without engagement there would be no space exploration to discover and interact with novel sentient beings across the galaxy.

## Star Trek’s Characters Versus State and Non-state Actors

In the 21st century, states are represented by national governments in jurisdictions that support the UN through formal commitments to respect and fulfill international treaties and resolutions. States are represented by elected or appointed government officials who interact with global and national non-state actors and actor networks^5-7^ to promote healthy and sustainable food systems. Unlike non-state actors, states are accountable for achieving their commitments and targets in the UN Decade of Action on Nutrition (2016-2025) and Sustainable Development Goals (SDGs) 2030 Agenda. In this commentary, Starfleet Officers are compared to states, and depicted as humans and other intergalactic species from various planets that correspond to jurisdictions, who are bound by political treaties and alliances through the Federation after decades of conflict, and who work together in shared diplomatic missions.^7,8,12^

Civil society organizations represent *Bajorans* who are admired for their traditions and spiritualism and* Klingons *who are warriors motivated by loyalty to defend virtuous causes. Academic institutions represent *Vulcans *and *Androids *as rational thinkers who process information systematically to solve problems.Commercial-sector entities are heterogeneous that represent *Humans;* shapeshifting *Changelings*; *Ferengi*motivated by profit but who provide business and entertainment structures that support the operations of the Federation and Starfleet;and *Romulans* and *Cardassians* who wield technical innovation and power to dominate other species.^7-12^ Industry trade organizations are portrayed as the *Borg* because they represent the collective interests of a network of actors in globalized food systems, and assimilate technological advancements and unique cultures and species.^7,12^ Star Trek explored how Starfleet engaged with and managed perpetual conflicts with the *Romulans, Cardassians,* and *Borg*.^7-12^

The Federation and the UN System each have principles and laws that govern how Starfleet and states, respectively, address challenges that affect citizens’ lives. The UN System has guidelines for states to ensure intersectoral, multi-stakeholder mechanisms to protect nutrition policies and programs from COI.^13,14^ The UN Decade of Action on Nutrition (2016-2025) recommended the need to “*Reconfigure global, national and subnational governance to ensure good governance mechanisms…be free from COI and coherently address all forms of malnutrition*.”^13^ The UN’s WHO and FAO have offered guidance to public service officials that emphasize their legal obligation to address COI and be held accountable for their actions.^1,13,14^

## Global Actors Who Responded to the WHO’s Consultation

Only six of 193 states participated in the WHO’s 2017 consultation including Brazil, Canada, Columbia and Namibia that supported the COI risk-assessment tool; New Zealand was mixed; and the United States was critical, which must be interpreted within the current political context. The US government and the Trump Administration have favored private-sector engagement to advance global health policy goals. Between 2017 and 2020, President Trump lowered the ethical standards of US public service accountability by accruing more than 3200 personal, business and financial COIs, establishing a pattern of political corruption and abuse of authority that has diminished US credibility as a member state.^15^ In 2020, President Trump withdrew financial support and terminated US membership in the WHO despite an ongoing coronavirus pandemic, a decision opposed by more than 750 US health and legal experts who advised that this action was unlawful and would harm national health and security interests.^16^

These events presented an opportunity for the WHO leadership to potentially disregard the US government’s comments about the COI risk-assessment tool. Engagement takes many forms including institutional indifference or opposition. Instead, the WHO Director General used diplomacy to confirm the important role of the United States to support the broader missions of the WHO and UN. Many Star Trek episodes depicted how Starfleet Officers challenged political corruption and unethical actions taken by governance bodies and alliances,^7,10-12^ which could inform how the UN and global nutrition and health governance actors respond to similar challenges.

## Motives and Actions of Commercial and Civil Society Actors

Commercial entities or private-sector actors have access to advanced technology, knowledge and financial resources to address malnutrition in countries worldwide to promote healthy and sustainable food systems.^4-6^ Commercial actors represent many Star Trek characters (ie, *Humans*, *Changelings *and *Ferengi ) *who provide beneficial business infrastructure and economic activities but that also produce consequences for the diets and health of populations.

Current evidence suggests that many private-sector actors (ie, *Romulans* and *Cardassians ) *have underperformed to achieve voluntary commitments, disregarded UN human rights best-practice guidelines for businesses, and engaged in predatory marketing practices that promote unhealthy food and beverage products that have fostered malnutrition and non-communicable diseases.^17^ All 14 commercial-sector entities that responded to the WHO’s consultation were either business or industry trade associations (ie, the *Borg*) that opposed the COI risk-assessment tool perceived as preventing partnerships.^3^ Alternative motives could be an inherent bias of these actors to prefer market-led solutions to address food system challenges, and that the COI tool would shift engagement power and decision-making back to states.

Civil society actors (*Bajorans* and *Klingons*) identify community needs, build and mobilize civic capacity, advocate for public- and private-sector commitments to address malnutrition, and ensure accountability for other actors’ actions.^18^ The 12 civil society actors who responded to the COI tool were mostly supportive.^3^ Certain civil society actors have suggested that commercial-sector actors have asserted their place at the global and national nutrition policy-making tables under the guise of multilateral food governance,^19^ and have cautioned the UN Secretary General to address COI in preparation for the UN Food Systems Summit in 2021.^19,20^ The World Economic Forum has been invited to participate and use multi-stakeholder market-led approaches to transform food systems.^21^

## Other Inter-Governance Actors

The Scaling Up Nutrition (SUN) Secretariat is a governance network established in 2010 that leads a global movement across 61 low- and middle-income countries to reduce malnutrition by 2030.^22^ This global governance body parallels Star Trek’s alliances and coalitions of planets established during the 22nd century that preceded the formation of the Federation in the 23rd century. SUN has been described as a global multi-stakeholder partnership, legitimized over the past decade using compelling narratives and institutional mechanisms to develop a reputation as a new leadership structure to address malnutrition.^23^ SUN has no official legal status but operates under the auspices of the UN Secretary General who appoints a leadership structure whose members include public and private donors.^23^ SUN has evolved in response to the UN System reforms intended to achieve the nutrition targets in the SDG 2030 Agenda.^24^

Ralston et al^3^ found that the SUN Secretariat had criticized the WHO’s risk-assessment tool perceived as restricting partnership engagement with states, but the SUN Secretariat’s UN Network supported the tool. Other SUN actors (ie, Civil Society, Donor, Business and Country Networks) did not participate in the WHO consultation. The UN Standing Committee on Nutrition recently merged with the SUN’s UN Network into UN Nutrition effective in 2021.^25^ It is unknown whether this change will harmonize the UN System approach to manage nutrition-related COI. Several UN agencies (ie, FAO, UNICEF and the World Food Programme) that influence nutrition policies did not respond to the consultancy,^3^ perhaps because each has its own engagement guidelines for non-state actors.

A 2020 SUN Movement strategic review identified several COI-related governance concerns.^26^ The review found that “*Experiences within and across SUN member countries show that ‘bringing people together’ … conducive to nutrition transformation requires due diligence around COI and the dynamics between constituencies*.”^26^ It also revealed concerns about *“Certain business and private-sector actors … perpetuating malnutrition through the marketing of and increased access to unhealthy processed foods” *in SUN countries linked to obesity and non-communicable diseases.^26^ The report recommended that the SUN Movement “*Needs to be more proactive in identifying and managing potential COI, in line with its principles of engagement to act with integrity and in an ethical manner and to do no harm*.”^26^ These findings are salient given decades of monitoring that demonstrate the ineffectiveness of states’ oversight of corporate practices that harm the nutrition and health of populations.^27^

## Moving Forward to Address Nutrition-Related COIs

In September 2019, the WHO held a consultation with representatives from 44 states who had pilot tested the risk-assessment tool.^28^ Most expressed support for the tool, which suggests momentum among state actors to re-establish their decision-making authority in the nutrition governance space.^28^ This observation aligns with the conclusion of Ralston et al that the WHO’s risk-assessment tool may promote policy coherence to address nutrition and diet-related non-communicable disease challenges.^3^ Case studies are available to understand how COIs manifest in national nutrition policies based on different political contexts.^29^ The UN has released resources for states to address COIs to foster productive partnerships to achieve the SDG 2030 Agenda,^30,31^ and the engagement stepsare remarkably similar to the WHO’s risk-assessment tool.

 Fifty years of Star Trek television and films reveal major insights to understand state and non-state actors’ motives, commitments and actions to address COI for nutrition policies and programs through global nutrition governance. First, engagement is inevitable for actors in a shared universe, there are many ways to engage, and without engagement there is no possibility of new discoveries. Star Trek’s Prime Directive is a non-interference engagement principle and moral commitment for Starfleet Officers to reduce harm, respect autonomy, and protect the rights of sentient beings who have self-awareness, intelligence and consciousness throughout the universe.

 The Prime Directive is not an absolute rule. Many episodes showed tensions within and between the Federation and Starfleet Command, and Starfleet Officers whose explorations required them to re-assess how to apply the Prime Directive principle when encountering new situations and interacting with novel sentient beings in the galaxy. Similarly, engagement principles are relevant to state and non-state actors who influence nutrition policies and programs. The WHO’s COI risk-assessment tool was intended to empower states to decide whether and how to engage with non-state actors to promote healthy and sustainable food systems. All actors must be held accountable when their actions undermine healthy and sustainable food systems.

Second, civil society or academic actors may use COI to justify non-engagement with commercial actors, which is appropriate when engagement has consequences for the diets and health of populations. But there are many governance challenges that are not necessarily COIs.^32^ In order to impact malnutrition in all forms, actors’ expressed and institutional commitments must be translated into operational and systemwide political commitments and actions.^4,33^ International treaties and resolutions, codes of conduct, principles and guidelines of inter-governance bodies, and laws can collectively be used to hold actors accountable for their actions^1,17^ to enable states to address the systematic nature of commercial influences,^34^ manage COIs and other governance challenges,^35^ and prevent the regulatory capture of states’ policy-making by commercial interests.^36^

 Finally, Star Trek’s stories and characters serve as allegories to understand power struggles among actors involved in global nutrition governance. Different forms of engagement are needed to manage conflicts among these actors who influence food systems and population health to achieve the commitments and targets in the UN Decade of Action on Nutrition (2016-2025) and SDGs 2030 Agenda on Earth in the 21st century.

## Acknowledgments

 I am grateful to Lina Mahy, Adrian Ng’asi, Victor Grech, and the anonymous reviewer for their insights and helpful feedback received on earlier versions of this commentary.

## Ethical issues

 Not applicable.

## Competing interests

 Author declares that she has no competing interests.

## Author’s contribution

 VIK is the single author of the paper.

## Disclosures

 VIK attended the 2015 WHO expert consultation and served as an independent consultant to WHO staff during 2016-2017 to develop the six-step risk assessment tool that was the focus of the online stakeholders’ consultation discussed in this paper.
